# Self-reported sleep disturbance in Crohn’s disease is not confirmed by objective sleep measures

**DOI:** 10.1038/s41598-020-58807-9

**Published:** 2020-02-06

**Authors:** Heba N. Iskandar, Emily E. Linan, Ami Patel, Renee Moore, Yi Lasanajak, C. Prakash Gyawali, Gregory S. Sayuk, Matthew A. Ciorba

**Affiliations:** 1 Emory University, Department of Medicine, Division of Digestive Diseases, Atlanta, Georgia USA; 2 Division of Gastroenterology Washington University School of Medicine, St. Louis, Missouri USA; 3 Duke University, Durham, North Carolina USA; 4 Rollins School of Public Health, Emory University, Atlanta, Georgia USA; 5 John Cochran Veterans Affairs Medical Center, St. Louis, Missouri USA

**Keywords:** Human behaviour, Crohn's disease

## Abstract

Sleep disturbance and fatigue are commonly reported among patients with Crohn’s disease (CD). In this prospective study, we aimed to define sleep quality in CD patients at various disease activity states and compare to healthy controls using objective and subjective measures. A prospective observational cohort study of CD patients seen at a tertiary academic inflammatory bowel diseases (IBD) clinic was compared to healthy volunteers. CD activity was assessed using the Harvey-Bradshaw Index (HBI). Sleep quality was assessed using the Pittsburgh Sleep Quality Index (PSQI) and Epworth Sleepiness Scale (ESS) and objectively over 1-week using actigraphy (motion-based) and morning urinary melatonin metabolite. 121 subjects (CD patients N = 61; controls N = 60) completed the study. 34 had active CD (HBI > 4). Sleep disturbance was more frequently reported by CD subjects than controls (PSQI: 57% vs. 35%, p = 0.02) and in patients with active CD versus in remission state (PSQI 75.8% vs. 33.3%, p < 0.01; ESS: 45.5% vs. 19%, p = 0.03). Sleep parameters as measured by actigraphy and urine melatonin metabolite did not vary by group. Crohn’s patients report significantly more disturbed sleep than controls. However, poor sleep was not confirmed by objective measures of sleep quality. Excessive daytime sleepiness in CD patients may be driven by factors beyond objectively measured poor sleep.

## Introduction

Crohn’s disease (CD) is one of the two major types of chronic inflammatory bowel disease (IBD) that together affect nearly 5 million individuals worldwide^1^. Fatigue is a common symptom associated with CD, particularly in patients with active disease. While the cause of fatigue can be multifactorial, sleep quality is considered a likely contributing factor^2^.

Several studies link inflammatory bowel disease, and CD in particular, with sleep disturbance and daytime sleepiness as well as fatigue^2–5^. The literature suggests a bidirectional dynamic to the cause-effect relationship of these disorders. Questionnaire-based studies have found that patients with active CD are more likely to have sleep disturbance than those in remission^2,5,6^. Alternatively, other questionnaire based studies indicate that sleep disturbance itself may place CD patients at increased risk of disease flares^7,8^. Animal studies confirm the latter by demonstrating that circadian disruption worsens colitis severity in experimental models^9^. This existing literature highlights the importance of this health issue; however most studies to date are limited by small patient cohorts or lack of objective sleep quality assessments or healthy controls for comparison.

To assess the relationship between CD and sleep we conducted a prospective cohort study examining both subjective and objective measures of sleep disturbance. Using these tools, we sought to determine whether CD diagnosis alone was associated with sleep disturbance and whether disease activity influenced sleep quality. Additionally, we examined a urinary metabolite of melatonin to determine if melatonin deficiency may exist as a plausible biological link between sleep disturbance and CD activity.

## Materials and Methods

### Study design and patient selection

We conducted a prospective cohort study examining sleep quality in CD patients and healthy controls. Eligibility criteria included patients with age range from 18–50 years. Crohn’s disease patients must have had an IBD specializing gastroenterologist confirm the CD diagnosis and been seen a minimum of two clinic visits. An age cutoff of 50 years was used to limit variability in sleep quality linked with aging^10^. Exclusion criteria for both CD and control subjects included factors that could impact sleep quality which were: functional GI disorders^11^, sleep apnea, BMI > 35, major medical illness (cardiopulmonary disorders, cirrhosis, or significant chronic renal disease or active malignancy), night-shift workers, smokers and heavy drinkers, and patients taking sedating medications (analgesics, muscle relaxants, antineoplastic agents, phenytoin, amphetamines, prescription weight-loss drugs, or benzodiazepines, thyroid medication, and anticholinergic/antihistamine medications)^12^.

CD patients were recruited from an outpatient gastroenterology clinic at Washington University School of Medicine in St. Louis. The medical charts of the CD patients were pre-screened to determine eligibility. Controls were recruited through a university-sponsored program supporting patient-oriented research (https://vfh.wustl.edu/). Eligibility for controls was initially assessed via a phone-based prescreen questionnaire that listed the exclusion criteria. Our hypothesis was that sleep disturbance is greater in CD patients than controls. Our aim was to recruit 60 subjects in both cohorts based on an assumed effect size of 0.51, using the actigraphy results from our IBS study (IBS: 63 +/− 43, Control: 98 +/− 90), an alpha level of 0.05, and a power of 80%^11^. We needed a sample size of 60 IBD patients and 60 controls for a final power of 80.1% to reject the null hypothesis.

### Patient assessments

All study participants completed comprehensive survey-based assessments and objective measurements of sleep quality at time of consent and/or while wearing the actigraph. The schedule of testing is detailed in Table 1. Demographics and relevant social history (smoking status, alcohol use, occupation, education level, housing and income) were also recorded. A list of complete comorbid illnesses for included subjects is included as Supplementary Table 1.

**Table 1:** Subjective and objective measures that were completed at time of consent and actigraphy.

Tests and procedures	Time of Consent		Time of Actigraphy													
	Healthy Controls	CD	Healthy controls							CD						
			Day 1	2	3	4	5	6	7	Day 1	2	3	4	5	6	7
Prescreened for eligibility	x	x														
Demographics and social history	x	x														
Pittsburgh Sleep Quality Index	x	x	x							x						
Epworth Sleepiness Scale	x	x	x							x						
Hospital Anxiety and Depression Scale	x	x	x							x						
Short Inflammatory Bowel Disease Questionnaire		x								x						
Short-form health survey	x	x	x							x						
Harvey Bradshaw index		x								x						
National Sleep Foundation Daily Sleep Log			x	x	x	x	x	x	x	x	x	x	x	x	x	x
Actigraphy			x	x	x	x	x	x	x	x	x	x	x	x	x	x
Urine sample									x							x

### Subjective assessments of sleep quality

Sleep quality was assessed using validated questionnaire-based assessments. Participants filled out the Pittsburgh Sleep Quality Index (PSQI) at the time of consent and actigraphy^13^. This questionnaire yields a global score and six component sub-scores related to subjective sleep quality, sleep latency, sleep duration, sleep efficiency, daytime sleepiness, sleep medications and sleep disturbances, as well as a global sleep score. Additionally, patients completed the Epworth Sleepiness Scale (ESS) at the time of consent and actigraphy^14^. ESS measures daytime sleepiness and the likelihood of falling asleep in eight different settings. All subjects completed sleep questions that were adapted from the National Sleep Foundation Daily Sleep Log (sleepfoundation.org/sleep-diary/SleepDiaryv6.pdf). Questions captured subjects subjective sleep quality. The sleep log allowed patients to record any information that may affect their sleep quality (napping habits, exercise, caffeine use, medication, GI daily symptoms and other environmental factors).

### Objective measures of sleep quality

Participants wore a motion logger actigraph (Ambulatory Monitoring, Inc. Ardsley, NY) continuously for a 7-day period. An actigraph is a watch like devices that records sleep patterns and circadian rhythms equivalent to a polysomnogram (PSG)^15,16^. Advantages of actigraphy as an objective measure of sleep quality include the ability to measure sleep quality in the patient’s natural environment for extended period of time^17^. The stored digital information from the actigraph was downloaded and analyzed using software (ActionW version 2.7.3045). Actigraphy data yielded total sleep time, latency to persistent sleep, sleep onset latency, total wake time during sleep, numbers of wake episodes, longest sleep episode duration, mean sleep episodes, and total sleep time (Supplementary Table 2). Actigraphy data was manually compared to the subject’s daily log by a single investigator trained by an experienced user and the software technicians to verify accuracy of the actigraphy software and identify any patient errors in use.

### CD activity

Assessment of clinical disease activity was performed in the clinic upon enrollment and at the end of the study period using the Harvey Bradshaw index (HBI)^18^. CD patients were grouped for dichotomous analysis with an HBI score of ≥5 considered to be active CD and those ≤4 as in remission^19^. Medications, disease severity and location of disease was based on the Montreal Classification were assessed by their physician at time of consent^20^.

### Quality of life measures

Three validated survey tools were used to assess subject quality of life. The Short Inflammatory Bowel Disease Questionnaire (SIBDQ) evaluates four domains of functioning: bowel symptoms, systemic symptoms, emotional symptoms and social factors^21^. For a generalized measure of comparison between control and CD patients, the Short-form 36 (SF-36) was used. The SF-36 is a validated assessment of health related quality of life which normalized to a maximum score of 100 with higher scores reflecting greater quality-of-life^22^. The Hospital Anxiety and Depression Scale (HADS) measures depression and anxiety with sub-scores classified as abnormal with a score ≥8^23,24^.

### Urine metabolite

The urinary 6-hydroxymelatonin sulfate/creatinine ratio (aMT6s:Cr ratio) correlates with nighttime circulating melatonin levels^24^. This biomarker is used as a surrogate biomarker in sleep studies examining outpatients not available for blood draws at awakening^25,26^. In this study, urine was collected in the early morning as the first morning void and refrigerated. Urine samples were sent via overnight FedEx (preaddressed and stamped) with a frozen icepack. Levels were previously demonstrated to be stable after overnight shipping for 24–48 hours^24^. Immediately upon receipt, samples were aliquoted and frozen at −80 °C for batched analysis by enzyme-linked immunoabsorbance assay (ELISA) (Genway Labs, SD, USA). The test results were normalized using creatinine concentration and expressed as nanograms of aMT6s per mg creatinine. Testing was performed as part of the scientific core service of the Yerkes Regional Primate Center biochemistry core at Emory University.

### Statistical analysis

Demographic, social, clinical characteristics, survey sleep measures and quality of life measures are reported utilizing mean (SD) for continuous variables and frequency (percentage) for categorical variables. The characteristics were compared in two different ways: 1) CD patients versus Control and 2) CD patients in remission versus those with active disease. For each of these comparisons, we compared means (medians) utilizing the independent two-sample t-test (or nonparametric Wilcoxon Rank-Sum test, as appropriate for non-normal distributions). We compared proportions utilizing Chi-square tests or the non-parametric Fisher’s exact tests. The aforementioned tests were also utilized to examine differences in Actigraphy measures between CD patients in remission and active CD patients. Multivariate logistic regression modeling was used to identify predictors of poor sleep (PSQI > 5). From the logistic regression model Odds Ratio (OR) and p-values are reported. Generalized linear regression and logistic regression were used to assess mediation^27^. Tests of hypotheses were two-sided, and a significance level of 0.05 was used throughout. All data analyses were performed using the statistical software package, SAS version 9.4

### Ethical considerations

The Human Research Protection Office (institutional review board) at Washington University School of Medicine approved this study and all subjects underwent the informed consent process. Our study was performed in accordance with all relevant guidelines and regulations. Our primary study data is available upon request.

## Results

### Patient characteristics

Medical records were pre-screened for eligibility criteria. A total of 178 CD patients were approached for enrollment during their physician visit. Of the 178 CD patients approached, 35 patients refused, 17 were ineligible for the study and 65 patients failed to follow-up with the study coordinator. A total of 110 Volunteer for Health participants contacted our study coordinator to serve as controls. 20 subjects were found ineligible by phone screen and 30 were lost to follow-up. A total of 121 subjects (CD patients N = 61; controls N = 60) completed the study. Of the CD patients, 34 were considered to have active disease (HBI > 4) during study participation. Participant demographics are listed in Table 2. Compared to the control cohort, enrolled CD patients were more likely to be of Caucasian race, married and carry current health insurance. Some CD patients listed a diagnosis of IBS that was given prior to CD diagnosis and this was not considered an exclusion criterion. Two patients, one CD patient and one control were diagnosed with a sleep disorder after enrollment. There were no significant differences in demographics between patients with CD in remission or active disease.

**Table 2:** Demographic, social and clinical characteristics of CD patients and controls.

Demographic and social characteristics	Control group (n = 60)	Crohn’s disease (n = 61)	P	Crohn’s disease/ Active (n = 34)	Crohn’s disease/remission (n = 27)	P
**Median Age (years)**	32	31.5	0.56	31.1	34.4	0.11
White	42 (71.2%)	53 (88.3%)		23 (85.2%)	30 (90.9%)	
Black	14 (23.7%)	7 (11.7%)		4 (14.8%)	3 (9.1%)	
Other	3 (5.1%)	0		0	0	
**Relationship status**			0.02			0.16
Never married	32 (53.3%)	26 (42.6%)		15 (55.6%)	11 (32.4%)	
Married	16 (26.7%)	30 (49.2%)		11 (40.7%)	19 (55.9%)	
Divorced, separated or widowed	12 (20.0%)	5 (8.2%)		1 (3.7%)	4 (11.8%)	
**Education**			0.93			0.96
High school or less	8 (13.3%)	7 (11.5%)		3 (11.1%)	4 (11.8%)	
Some college	19 (31.7%)	19 (31.2%)		9 (33.3%)	10 (29.4%)	
College degree	23 (38.3%)	22 (36.1%)		10 (37%)	12 (35.3%)	
Graduate degree	10 (16.7%)	13 (21.3%)		5 (18.5%)	8 (23.5%)	
**Family income**			0.77			0.46
<$20,000	10 (16.7%)	7 (12.1%)		3 (12%)	4 (12.1%)	
$20,000–49,999	16 (27.1%)	18 (31.0%)		7 (28%)	11 (33.3%)	
$50,000–99,999	23 (39.0%)	19 (32.8%)		6 (24%)	13 (39.4%)	
$100,000–149,999	5 (8.5%)	6 (10.3%)		4 (16%)	2 (6.1%)	
Chose not to answer	5 (8.5%)	8 (13.8%)		5 (20%)	3 (9.1%)	
**Employment:** Employed/in school	49 (87.5%)	54 (90.0%)	0.67	25 (92.6%)	29 (87.9%)	0.68
**Housing:** Rent or Own	52 (96.3%)	57 (98.3%)	0.61	25 (96.2%)	32 (100%)	0.45
**Insured**	47 (81.0%)	58 (95.1%)	0.02	25 (92.6%)	33 (97.1%)	0.58
**Current tobacco use**	2 (3.4%)	5 (8.3%)	0.44	3 (11.5%)	2 (5.9%)	0.64
BMI* (kg/m^**2**^**)**	25.5 (4.6)	25.4 (4.1)	0.9	24.9	25.8	0.26
IBS diagnosis**	1 (1.7%)	14 (23.3%)	<0.01	7 (21.2%)	7 (25.9%)	0.67
**Sleep disorder**	0	1 (1.6%)	1	0	1 (3.7%)	0.44
**Current medications**						
antiTNF***	0	42 (71.2%)	<0.01	23 (75%)	18 (66.7%)	0.48
Immunomodulators	0	41(68.3%)	<0.01	21 (63.6%)	20 (74.1%)	0.39
ASA	0	18 (30.5%)	<0.01	11 (34.4%)	7 (25.9%)	0.48
Prednisone	0	3 (5.1%)	0.25	3 (9.4%)	0	0.24
Antidepressant	4 (7.7%)	11 (18.6%)	0.1	9 (28.1%)	2 (7.4%)	0.05

*BMI: body mass index, **IBS: irritable bowel syndrome, ***antiTNF: anti-tumor necrosis factor.

### Subjective measures of sleep

Subjective assessments of sleep and quality of life scores are provided in Table 3. By PSQI questionnaires, CD patients were significantly more likely to have disturbed sleep than controls (57% vs 35%, p = 0.02). This effect was driven by patients with active CD. In subgroup analysis, sleep disturbance was significantly more pronounced in patients with active CD (HBI > 4) than in patients with CD in remission (75.8% poor sleep in the active CD group vs. only 33.3% in patients in remission, p < 0.01). This effect also held true in the ESS (45.5% poor sleep in active CD vs. 19% in CD in remission, p = 0.03). Not surprisingly, patients with active CD had a poorer quality of life, as measured by SIBDQ and SF-36.

**Table 3:** Subjective sleep measures and quality of life measures, comparing study groups.

	Crohn’s disease (n = 61)	Control group (n = 60)	P	Crohn’s disease, remission (n = 27)	Crohn’s disease, active (n = 34)	P
ESS (>10)	20(33.9%)	17(30.9%)	0.73	5(19.2%)	15(45.5%)	0.03
PSQI (>5)	34(56.7%)	21(35%)	0.02	9(33.3%)	25(75.8%)	<0.01
**HADS**						
*Anxiety (>8)*	25(41.7%)	16(26.7%)	0.08	10(37%)	15(45.5%)	0.51
*Depression (>8)*	5(8.3%)	4(6.7%)	0.75	2(7.4%)	3(9.1%)	0.75
SIBDQ (≥50)	NA	NA	NA	26(96.3%)	11(33.3%)	<0.01
SF36	70.1	79.6	<0.01	77	64.4	<0.01
#bm/day*	2.9	1.3	<0.01	1.8	3.8	<0.01
#bm/night*	0.4	0.1	<0.01	0.1	0.7	0.01

*Provided in Table 3 as included in logistic regression analysis.

#bm: number of bowel movements.

We characterized predictors of poor sleep in the Crohn’s patients as measured by PSQI using a multivariable logistic regression model (Table 4). We also addressed confounders of CD activity as a variable predicting poor sleep. Significant predictors of poor sleep included active CD (OR 3.6, p = 0.03), anxiety (OR 1.3, p < 0.01), and higher BMI (OR 1.2, p = 0.01). A higher urine melatonin level approached significance as a predictor associated with poor sleep (OR 1.01, p = 0.05). This model is adjusted for IBS diagnosis, anti-TNF therapy, antidepressant use, depression using HADS score, SIBDQ, number of bowel movements, and sleep onset latency. Number of bowel movements interrupting sleep was included as a potential confounder. Even though study participants in the CD group have significantly more bowel movements (Table 3), this variable was not significant in our multivariable logistic regression model.

**Table 4:** Predictors of poor sleep as measured by PSQI* among all patients included using multivariable logistic regression**.

Predictor Variable	OR	95% CI	p
Active Crohn’s vs. Crohn’s in remission and controls	3.6	1.1–11.4	0.03
BMI	1.2	1.0–1.3	0.01
Anxiety as measured by HADS	1.3	1.1–1.5	0.001
Urine Melatonin	1.01	1.0–1.02	0.05

*Poor sleep defined as PSQI > 5.

The association of poor sleep by PSQI and the following variables were accessed using logistic regression modeling (Crohn’s disease status active vs (control + remission), IBS diagnosis, anti-TNFα therapy, antidepressant use, BMI (cont.), HADS_anxiety2(cont.), HADS_depression2(cont.), SIBDQ(cont.), BowlMovementPerDay(cont.), PatientReportedSleepLatency(cont.), Urine Melatonin(cont.).

### Objective measures of sleep

The subjective questionnaires results were in contrast with the objective actigraphy data. Actigraphy data showed no significant differences in sleep measures including total sleep time, sleep onset latency, sleep efficiency, and total waking time during sleep, between control and CD patients or between CD in remission compared with active CD (Table 5).

**Table 5:** Actigraphy results, comparing CD severity groups and controls.

	Crohn’s Disease (n = 61)	Control group (n = 60)	P	Crohn’s disease, remission (n = 27)	Crohn’s disease, active (n = 34)	P-value*
Total sleep time (min)	405.8	415.8	0.15	408	403.9	0.93
Sleep efficiency	91%	91.50%	0.64	92.20%	90.10%	0.19
Latency to persistent sleep (min)	9	9	0.8	8.5	9.5	0.29
Total wake time during sleep (min)	50	48.2	0.53	48.8	50.9	0.5
Numbers of wake episodes	7.8	8.5	0.23	7.3	8.2	0.35
Longest sleep episode duration (min)	209.1	213.1	0.8	222	197.9	0.34
Mean sleep episode duration (min)	91.6	80.3	0.32	103.9	80.8	0.25
Percentage of subjects with normal total sleep time	72.4%	84.20%	0.13	77.80%	67.70%	0.39
Percentage of subjects with normal sleep efficiency	86.4%	85.70%	0.65	92.60%	81.30%	0.19
Percentage of subjects with abnormal wake time during sleep	51.7%	42.90%	0.34	44.40%	57.60%	0.31

**Figure 1:**
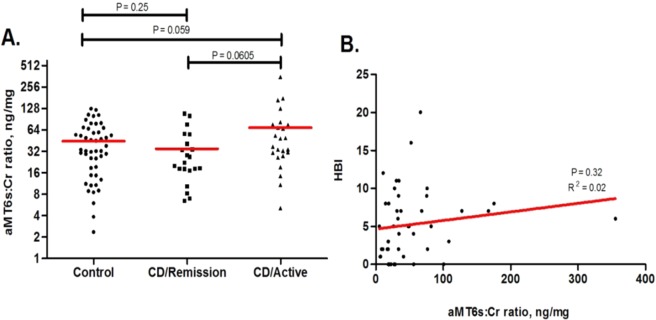
Urinary 6-hydroxymelatoin sulfate/creatinine ratio (aMT6:CR ratio) comparing study groups and correlation between Harvey Bradshaw Index (HBI) and a Mt6s:Cr ratio.

Urine melatonin was not significantly different between study groups, and there was no significant correlation between melatonin and disease activity (p = 0.09 for active CD vs the other 2 groups, and p = 0.14 across the 3 groups). Hence, the magnitude of the effect of urine melatonin on sleep quality decreases when adjusting for CD activity (Fig. 1).

**Figure 2:**
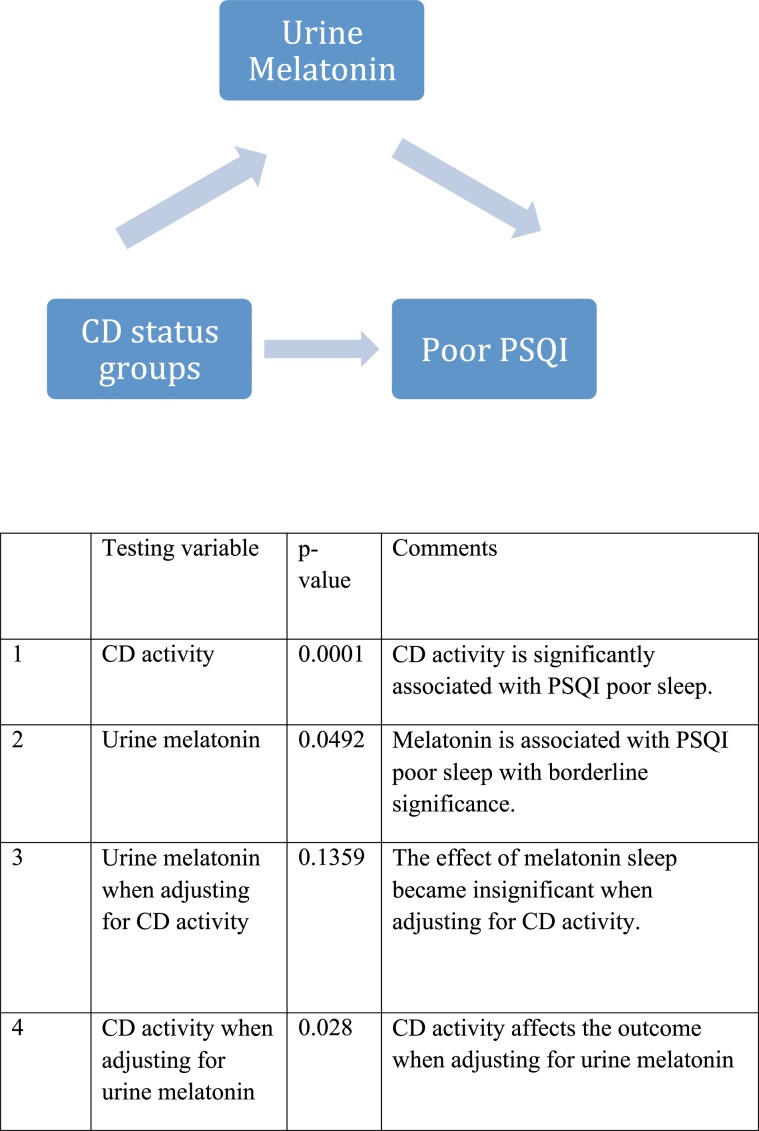
Mediation effect of Urine melatonin on Crohn’s disease status (active vs (control + remission)) in predicting PSQI poor outcome (PSQI_2 > 5) using logistic regression modeling.

In order to evaluate the relationship between melatonin and sleep in our study population, we utilized mediation analysis. Active CD (compared to controls and patients in remission) is significantly associated with poor sleep quality as measured by PSQI, even when adjusting for urine melatonin levels (p = 0.03). Urine melatonin had no mediation effect on the causal relationship between disease status and sleep quality (Fig. 2).

## Discussion

In this large study, we comprehensively applied a real-time quantitative measurement tool (actigraphy) and validated sleep questionnaires to define sleep quality in patients with CD in different states of disease activity and as compared to healthy controls. This is the first study of its kind with a control population and to include melatonin metabolites as a biologic correlate of sleep quality in this population. In composite, CD patients report significantly disturbed sleep as compared controls on subjective questionnaires, but this is not confirmed by quantitative sleep measurement by actigraphy. Moreover, urine melatonin metabolite levels were also not significantly different between groups. These results indicate that daytime sleepiness and poor sleep as reported in IBD patients may be driven by factors beyond objectively measured poor sleep.

The perception of disturbed sleep has been evaluated using a variety of survey measures (ESS, PSQI and PROMIS). In past studies using these measures, there has been considerable support for disturbed sleep in patients with inactive and active disease, when compared to controls^2,3,8^. Our subjective data builds on previous data showing that sleep disturbances exist in CD patients compared to controls and this difference is more pronounced with active CD patients. A survey study utilizing PSQI has shown a correlation of poor sleep to mucosal healing in small number of patients, despite clinical remission^28^. An additional study using electroencephalography showed more features of disturbed sleep in 8 pediatric patients with active IBD compared to 14 patients with IBD in remission and 24 healthy controls^29^. Furthermore, elevated inflammatory markers (C-reactive protein) in the absence of night symptoms were significantly associated with subjectively assessed poor sleep in IBD patients^30^.

Our objective data (actigraphy), however, is counter to what other studies and our hypothesis have suggested. A further characterization of sleep disturbance shows that CD patients have significantly more daytime sleepiness. Daytime sleepiness was found to be important in subjective questionnaires, which suggests that patients have the strong perception of falling asleep especially during the day. IBD patients commonly also report fatigue as a primary concern. Despite the correlation between fatigue and diminished QOL, doctors lack the robust knowledge on the pathogenesis of IBD-related fatigue. Fatigue is caused by a complex interaction between medication side effects, poor nutrition, depressive symptoms and inflammation, among others. Other survey-based studies have correlated sleep disturbance in IBD patients to fatigue^2,31^. A systematic approach is required to treat fatigue in patients with IBD, which has a sizeable chance of improving QOL for our IBD patients.

We evaluated possible contributors to the difference between subjective and objective sleep measures in our study. While our study was not designed to evaluate fatigue, a post-hoc analysis within our SIBDQ questionnaires revealed that patients with active CD reported fatigue more commonly than patients with disease in remission (80% of active CD patients have fatigue vs. 44% of patients with inactive CD, Fisher’s exact p = 0.007). Independent of quantitatively objective poor sleep, more CD patient’s scores on the ESS measuring daytime sleepiness may be driving the patient’s perception of poor sleep (Table 4). While fatigue and daytime sleepiness are separate, they may overlap in the same patients with active disease and affect our patients’ survey responses. Future work would measure fatigue and evaluate these two important measures and their possible link in CD.

Participants wore an Actigraph (Ambulatory Monitoring, Inc.) on their wrist for one week. Actigraphy is a validated technology that has been shown in many situations to be correlated to PSG in determining subjects’ sleep duration and awakenings from sleep^15^. While PSG has advantages over actigraphy in establishing sleep disorders, actigraphy is a good representation of a patient’s sleep over time and allows patients to sleep in their normal environment. Nevertheless, actigraphy is dependent on patient adherence. We acknowledge that patients could be harboring sleep disorders and certain diagnoses that can only be established via polysomnography, but are confident in the advantages of actigraphy including its practical administration override the downfalls for this type of real-world observational study as shown in peer-reviewed studies^16,17^.

Limitations exist in the current literature addressing sleep and IBD health and our study. The majority of studies use only subjective self-reported patient questionnaires to assess sleep quality^2,5,8^. While these subjective sleep surveys are validated with objective measures in the general population, only one other study has utilized an IBD population or one that includes active disease^3^. Previous studies using objective sleep assessments such as melatonin and polysomnography were performed only in small IBD patient cohorts in remission^3,32^. The largest study to date examining this relationship was part of a larger IBD patient health study and thus had more limited assessments of sleep quality^8^. Another interesting premise is the idea that poor sleep can lead to increased IBD activity as suggested by animal models, hence the directionality of the relationship between poor sleep/fatigue and active Crohn’s is hard to assess^33^. Actigraphy has been used in one other large study of CD patients. Qazi *et al*. evaluated 72 patients with CD who were stratified by HBI into remission, mild and moderate-to-severe disease^34^. While this study lacked a control population, they found no difference in total sleep time or sleep latency, but found that patients with moderate-to-severe disease had reduced sleep efficiency and more minutes awake after falling asleep. On multivariate analysis only more minutes awake was significant in the moderate-to-severe CD group and controlled substance use (marijuana and opiate) was associated with poor sleep efficiency. A less strict set of exclusion criteria including the enrollment of patients on marijuana and opiates may account for the observed differences in this study.

We only used self-reported measures of CD disease activity. We acknowledge the limitations of using only self-reported survey measures of CD activity as opposed to objective measures such as endoscopy and fecal calprotectin and its possible effect on our comparison between active and inactive CD. Furthermore, we included 11 patients (18.6%) in our Crohn’s group who are on antidepressants as well as 4 patients (7.7%) in the control group, given the real-world common use of these medications. Given that this could affect sleep, we adjusted for this variable of antidepressant use in our multivariate analysis. Future directions in relation to this work could include incorporating specific fatigue measures and questionnaires, attentiveness scales, and using commercially available HR monitors that would decrease the cost and difficulty of further study.

Tryptophan metabolism is an important pathway in the pathophysiology of both sleep disturbance and depression^35^. Serotonin and melatonin, neurotransmitters involved in mood and sleep regulation respectively, are direct metabolic products of tryptophan along the same pathway. In active Crohn’s disease, tryptophan metabolism is instead shunted away from these metabolites along the kynurenine pathway^36^. In our study, there somewhat unexpectedly a trend toward lower urinary melatonin metabolite levels in controls compared to CD patients and no differences in melatonin based on CD activity. As tryptophan metabolism can be modified at many levels, compensatory melatonin secretion may help intrinsically correct or treat poor sleep when disease is active. It is however noted that the use of the urinary melatonin metabolite and melatonin as a marker of sleep health is limited by variations due to age, seasons of the year, light exposure duration and intensity, shift work, serious illness, and diet, as well as genetic factors^37–39^.

In conclusion, the perception of sleep disturbance in CD patients is an important health issue that should be addressed. Poor sleep and the perception of daytime sleepiness in active CD are not confirmed by objective measures (actigraphy) of sleep disturbance in this study cohort. Patient-reported outcomes are at the forefront of treatment goals in CD, and further investigation into the reasons self-reported poor sleep in CD is warranted. As poor sleep is correlated to fatigue, future studies on the mechanisms of fatigue in CD are also needed due to the intriguing relationship between sleep and fatigue.

## Supplementary information

Supplementary Tables

## Data Availability

The datasets generated during the current study are available from the corresponding author on reasonable request.
