# Supplementary material for: Self-reported sleep disturbance in Crohn’s disease is not confirmed by objective sleep measures
Source: Sci Rep. 2020 Feb 6;10:1980. doi: 10.1038/s41598-020-58807-9 (PMC7005285; doi:10.1038/s41598-020-58807-9)
Supplement: Supplementary file 1 — Supplementary Tables [file 41598_2020_58807_MOESM1_ESM.docx]

Supplementary material

Self-reported sleep disturbance in Crohn’s disease is not confirmed by objective sleep measures

Heba N Iskandar, MD^1,2*^

Emily E Linan^1*^

Ami Patel^1^

Renee Moore^3^

Yi Lasanajak^3^

C. Prakash Gyawali, MD^1^

Gregory S. Sayuk MD, MPH^1,4^

Matthew A Ciorba MD^1^

**Supplementary Table 1. Medical co-morbidities of our study participants***

| Co-morbidities | **Crohn’s group (n=61)** | **Control group (n=60)** |
| --- | --- | --- |
| Anxiety/Depression | 11 | 4 |
| Seasonal allergies | 4 | 3 |
| Osteoporosis/osteopenia | 2 | 0 |
| Hypertension | 2 | 1 |
| GERD | 5 | 1 |
| Osteoporosis/osteopenia | 2 | 0 |
| Migraines | 1 | 0 |
| Hypothyroidism | 2 | 1 |
| Mild asthma | 1 | 2 |
| Well-controlled type II DM | 1 | 0 |

***# of patients with each condition; no statistically significant differences**

**Supplementary Table 2. Definitions of actigraphy parameters**

| **Actigraphy Definitions** | |
| --- | --- |
| **Total sleep time** | Duration of all sleep minutes scored as sleep |
| **Sleep efficiency** | % of time patient spend sleeping from sleep onset to sleep offset |
| **Latency to persistent sleep** | ratio of sleep time between first continuous block of 20 minutes sleep and sleep offset |
| **Total wake time during sleep** | Total minutes scored as wake |
| **Numbers of wake episodes** | # of contiguous wake epochs |
| **Longest sleep episode duration** | Duration of sleep episodes in minutes. |
| **Mean sleep episode duration** | Mean duration of contiguous sleep epochs in minutes |
| **Percentage of subjects with normal total sleep time** | % of patients with total sleep time over 360 minutes |
| **Percentage of subjects with normal sleep efficiency** | % of patients sleeping 85% to 100% of the time from sleep onset and offset |
| **Percentage of subjects with abnormal wake time during sleep** | % of patients that are awake for 30 minutes or more during sleep episodes |
